# Introduction to Alcohol Withdrawal

**Published:** 1998

**Authors:** Richard Saitz

**Affiliations:** Richard Saitz, M.D., M.P.H., is an assistant professor of medicine in the Clinical Addiction Research and Education Unit, Section of General Internal Medicine, Department of Medicine, at Boston Medical Center and Boston University School of Medicine; a faculty fellow in the Center for Substance Abuse Prevention Faculty Development Program; and a generalist physician faculty scholar of the Robert Wood Johnson Foundation

**Keywords:** AOD withdrawal syndrome, biochemical mechanism, neurotransmission, neurotransmitter receptors, central nervous system, symptom, tremor, anxiety state, delirium tremens, AODR (alcohol and other drug related) hallucinosis, AODR seizure, AOD abstinence, disease severity, patient assessment, treatment method, alcohol withdrawal agents, drug therapy, detoxification, addiction care, literature review

## Abstract

Heavy drinkers who suddenly decrease their alcohol consumption or abstain completely may experience alcohol withdrawal (AW). Signs and symptoms of AW can include, among others, mild to moderate tremors, irritability, anxiety, or agitation. The most severe manifestations of withdrawal include delirium tremens, hallucinations, and seizures. These manifestations result from alcohol-induced imbalances in the brain chemistry that cause excessive neuronal activity if the alcohol is withheld. Management of AW includes thorough assessment of the severity of the patient’s symptoms and of any complicating conditions as well as treatment of the withdrawal symptoms with pharmacological and nonpharmacological approaches. Treatment can occur in both inpatient and outpatient settings. Recognition and treatment of withdrawal can represent a first step in the patient’s recovery process.

Every year more than one-and-a-half million people in the United States either enter alcoholism treatment or are admitted to a general hospital because of medical consequences resulting from alcohol dependence. These patients, as well as a substantial number of other people who stop drinking without seeking professional treatment, experience alcohol withdrawal (AW). AW is a clinical syndrome that affects people accustomed to regular alcohol intake who either decrease their alcohol consumption or stop drinking completely. In these people, the central nervous system (CNS) has adjusted to the constant presence of alcohol in the body and compensates for alcohol’s depressive effects on both brain function and the communication among nerve cells (i.e., neurons). Consequently, when the alcohol level is suddenly lowered, the brain remains in a hyperactive, or hyperexcited, state, causing withdrawal syndrome.

AW syndrome varies significantly among alcoholics in both its clinical manifestations and its severity. These manifestations^1^ can range from mild insomnia to severe consequences, such as delirium tremens (DT’s) and even death. Substantial variability also exists in the incidence with which symptoms occur in various drinkers. Some people who regularly consume alcohol never experience any withdrawal symptoms. Conversely, in some alcoholics withdrawal symptoms can occur at blood alcohol concentrations (BAC’s) that would be intoxicating in non-alcohol-dependent people but which for the dependent patients represent a decline from their usual BAC’s.

This article briefly reviews the mechanisms, clinical features, and management of AW. The article also discusses how the treatment of AW can be linked to the treatment of alcohol dependence and any co-occurring or underlying disorders. For more in-depth discussions of some of these issues, the reader is referred to subsequent articles in this issue.

## Mechanisms of Alcohol Withdrawal

Historically, several mechanisms have been suggested to play a role in the development (i.e., etiology) of AW. For example, researchers initially thought that withdrawal might be caused by nutritional deficiencies (Isbell et al. 1955; Victor and Adams 1953) and that some complications of withdrawal (e.g., seizures) might result directly from alcohol use or intoxication (Ng et al. 1988). Although alcoholic patients exhibit many metabolic and nutritional disturbances, overwhelming laboratory and clinical evidence now indicates that the constellation of signs and symptoms known as AW are caused by interrupting the constant exposure of the CNS to alcohol.

The hypothesis that withdrawal occurs as a result of “insufficient” alcohol intake or abstinence in dependent patients rather than because of nutritional deficiencies was supported by an early study of men who received large daily doses of alcohol (Isbell et al. 1955). The study participants, who also were well fed, each consumed up to almost 30 standard drinks per day for up to 3 months. Upon abstaining from this alcohol intake, these men invariably developed withdrawal symptoms. Moreover, the symptoms of AW were dose dependent: The men who had consumed the largest amounts of alcohol developed the most severe manifestations of withdrawal, such as hallucinations, seizures, and DT’s.

These findings support the association between alcohol intake and the clinical manifestations of withdrawal syndrome.

To better understand the mechanisms underlying withdrawal, one must briefly review some of the principles of neuronal communication in the CNS. The transmission of nerve signals from one neuron to the next is achieved, in general, through small molecules called neurotransmitters, which are secreted by the signal-emitting neuron. The neurotransmitter molecules traverse the small gap (i.e., the synapse) between adjacent neurons and interact with docking molecules (i.e., receptors) on the signal-receiving neuron. The interaction between a neurotransmitter and its receptor initiates a cascade of chemical and electrical reactions in the signal-receiving cell that depending on the neurotransmitter involved, results in the activation or inhibition of that cell. Thus, excitatory neurotransmitters (e.g., glutamate) stimulate the signal-receiving neuron, whereas inhibitory neurotransmitters (e.g., gamma-aminobutyric acid [GABA]) inhibit the neuron. Under normal conditions, a tight balance is maintained between excitatory and inhibitory influences.

Regular alcohol intake affects numerous excitatory and inhibitory neurotransmitter systems in the brain (Begleiter and Kissin 1996). Similarly, many neurotransmitters and mechanisms probably are involved in AW. Of these neurotransmitters, scientists best understand the roles of GABA and glutamate. For example, researchers have demonstrated that alcohol enhances (i.e., potentiates) GABA’s inhibitory effects on signal-receiving neurons, thereby suppressing neuronal activity. With chronic alcohol exposure, however, GABA receptors become less responsive to the neurotransmitter, and higher alcohol concentrations are required to achieve the same level of suppression. This clinically observed adaptation is referred to as tolerance.

When alcohol is removed from this adapted system, the GABA receptors remain less responsive, leading to an imbalance in favor of excitatory neurotransmission. This imbalance is enhanced further by an alcohol-induced increase in the number of one type of receptor for the excitatory neurotransmitter, glutamate. Even when alcohol is removed, the number of these receptors remains elevated, leading to enhanced excitatory neurotransmission. Both of these mechanisms contribute to the neuronal hyperexcitability that is characteristic of AW. (For more information on the neurochemical mechanisms underlying withdrawal, see the article by Littleton, pp. 13–24.)

## Clinical Features of Alcohol Withdrawal

Despite this current understanding of the mechanisms underlying AW syndrome, some controversies still exist regarding the risk, complications, and clinical management of withdrawal. These controversies likely arise from the varied clinical manifestations of the syndrome in alcoholic patients and from the diverse settings in which these patients are encountered. For example, some alcoholic patients who cut down or stop drinking may experience no withdrawal symptoms, whereas others experience severe manifestations. In fact, even in clinical studies of patients presenting for alcohol detoxification, the proportion of patients who developed significant symptoms ranged from 13 to 71 percent (Victor and Adams 1953; Saitz et al. 1994). What is the reason for this variability? Likely, individual patients differ in their underlying risks for withdrawal symptoms. These differences result from factors such as the patient’s pattern of alcohol use, the presence of coexisting illnesses, variations in genetic influences and CNS mechanisms, as well as the neurochemical mechanisms described in the previous section.

Despite the variability in the type and severity of symptoms that a person can experience, the clinical syndrome of AW has been well defined. Its symptoms generally appear within hours of stopping or even just lowering alcohol intake and, thus, BAC. The most common symptoms include tremor, craving for alcohol, insomnia, vivid dreams, anxiety, hypervigilance,^2^ agitation, irritability, loss of appetite (i.e., anorexia), nausea, vomiting, headache, and sweating. Even without treatment, most of these manifestations will usually resolve several hours to several days after their appearance.

The most severe manifestations of AW include hallucinosis, seizures, and DT’s (see also the figure on pp. 63, from Victor and Adams’ classic paper).

Hallucinosis, which may occur within 1 or 2 days of decreasing or abstaining from alcohol intake, is a complication distinct from DT’s. Patients with alcohol hallucinosis see, hear, or feel things that are not there even though they are fully conscious and aware of their surroundings. Moreover, hallucinosis is not necessarily preceded by various physiological changes (i.e., autonomic signs).

AW seizures also can occur within 1 or 2 days of decreased alcohol intake, even in the absence of other withdrawal signs and symptoms. The patient usually experiences only one generalized convulsion, which involves shaking of the arms and legs and loss of consciousness. If a second convulsion occurs, it generally happens within 6 hours of the first seizure (Victor and Brausch 1967). Although multiple seizures are not common, AW is one of the most common causes in the United States of status epilepticus—a medical emergency characterized by continuous, unrelenting seizures.

DT’s, which last up to 3 or 4 days, are characterized by disorientation and are usually accompanied by autonomic signs resulting from the activation of the nerves responsible for the body’s response to stress). Those signs include severe agitation, rapid heartbeat (i.e., tachycardia), high blood pressure, and fever. (Thus, DT’s are a much more serious condition than the “shakes,” which often are also colloquially referred to as DT’s.) DT’s can develop between 1 and 4 days after the onset of withdrawal and are generally preceded by additional autonomic signs, such as sweating and tremors. About five percent of the patients who experience DT’s die from metabolic or cardiovascular complications, trauma, or infections (Victor and Adams 1953; Cutshall 1964).

### Risk Factors for DT’s and Seizures

Given the wide range of potential manifestations associated with withdrawal, is it possible to predict their development in individual patients? Currently, the answer is “no.” To date, most studies of predictors of severe or complicated withdrawal have been too limited methodologically to allow clinically accurate prognoses for individual patients. Based on current understanding of the withdrawal syndrome, as well as on some clinical research results, however, clinicians have identified some patient characteristics that likely confer a risk of more severe withdrawal symptoms; prolonged symptoms; or withdrawal-specific complications, such as DT’s or seizures. These factors include the following:

- More severe alcohol dependence, including prior development of withdrawal symptoms
- Higher levels of alcohol intake, resulting in higher BAC’s
- Longer duration of alcoholism
- Abnormal liver function
- Prior detoxification
- Past experience of seizures or DT’s
- Intense craving for alcohol
- Concomitant acute illness
- Older age
- Use of other drugs in addition to alcohol
- More severe withdrawal symptoms when presenting for treatment.

## Management of Alcohol Withdrawal

### Assessment

Before initiating any interventions, the first step in managing a patient’s withdrawal is to assess thoroughly the patient’s condition. This assessment should include an evaluation of the presence of coexisting medical and psychiatric conditions, the severity of the withdrawal symptoms, and the risk of withdrawal complications. Moreover, reassessment of the withdrawal symptoms at regular intervals until they have resolved can help guide treatment as well as encourage the clinician to monitor the patient for the development of complications that might require more intensive observation or treatment.

The symptoms of withdrawal are not specific and easily can be confused with other medical conditions. Consequently, the clinician’s initial assessment also serves to exclude other conditions with symptoms similar to those of AW. Examples of such conditions include subdural hematoma (i.e., the collection of blood in the space between the membranes surrounding the CNS), pneumonia, meningitis, and other infections. Similarly, seizures and DT’s may be confused with other conditions that should be excluded during initial assessment. For example, DT’s, which represent an acute confusional state, can mimic delirium from other medical causes, such as encephalitis, meningitis, adverse effects of some medications, or Wernicke’s encephalopathy.^3^ Likewise, AW seizures must be distinguished from seizures resulting from other causes, such as mineral or electrolyte abnormalities, strokes, brain tumors, epilepsy, or subdural hematoma. Thus, a diagnosis of DT’s and AW seizures should be made only after other reasonable causes for these complications have been excluded.

A thorough assessment also should anticipate health problems that frequently occur in patients withdrawing from alcohol. These complications may include the following:

- Gastritis (i.e., an inflammation of the stomach lining, which often is associated with bleeding)
- Gastrointestinal bleeding (e.g., from the esophagus, stomach, or intestines)
- Liver disease
- Cardiomyopathy (i.e., any disorder of the heart muscle)
- Pancreatitis (i.e., an inflammation of the pancreas)
- Disturbances in the electrolyte balance (e.g., alcohol ketoacidosis—a metabolic derangement that results in too much acid in the bloodstream—and abnormally low levels of magnesium in the blood)
- Deficiency of the vitamin folate, which can cause lower-than-normal numbers of blood cells
- Deficiency of the vitamin thiamine, which can lead to serious neurological problems, such as Wernicke’s encephalopathy (accordingly, thiamine should be administered to all patients undergoing AW to prevent the development of this syndrome).

**Figure 1:**
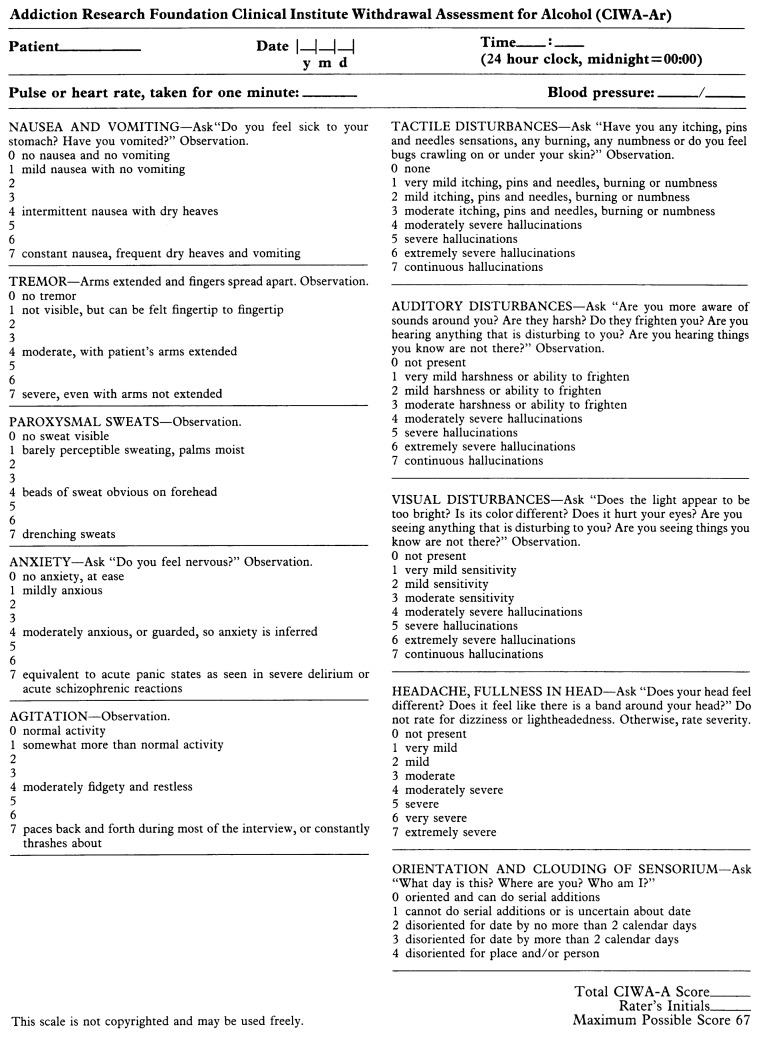
The Clinical Institute Withdrawal Assessment for Alcohol, revised (CIWA–Ar) (Sullivan et al. 1989; Foy et al. 1988). This instrument rates 10 withdrawal features, takes only a few minutes to administer, and can be repeated easily when necessary. A total score of 15 or more points indicates that the patient is at increased risk for severe withdrawal effects, such as confusion and seizures.

Once a diagnosis of AW has been made, the clinician must assess the severity of withdrawal and the risk for associated complications. The best validated tool for such an assessment is the Clinical Institute Withdrawal Assessment for Alcohol, revised (CIWA–Ar) (Sullivan et al. 1989; Foy et al. 1988) (see figure 1). This instrument, which rates 10 withdrawal features, can be administered in only a few minutes and repeated when necessary. A total score of 15 or more points indicates that the patient is at an increased risk for confusion and seizures.

### Treatment of Alcohol Withdrawal

Based on the patient’s score on the CIWA–Ar, the physician determines the appropriate treatment (see table). For all patients, especially those experiencing severe withdrawal symptoms, proven benefits of treatment include amelioration of symptoms, prevention of both seizures and DT’s, and treatment of DT’s. Treatment also may prevent increasing severity of withdrawal during subsequent withdrawal episodes and encourage the patient to enter alcoholism treatment for relapse prevention.

Patients with mild withdrawal symptoms (i.e., CIWA–Ar scores of 8 or less) and no increased risk for seizures can be managed without specific pharmacotherapy (Mayo-Smith 1997; Saitz and O’Malley 1997). Successful nonpharmacological treatments include frequent reassurance and monitoring by treatment staff in a quiet, calm environment. Most patients with mild withdrawal symptoms, whether they are treated or not, do not develop complications.

**Table:** Examples of Specific Regimens Used in the Treatment of Alcohol Withdrawal

Treatment Approach	Treatment Component
Monitoring	Monitor the patient by administering the CIWA–Ar^1^ test every 4 to 8 hours until the score has been lower than 8 to 10 points for 24 hours Use additional assessments as needed
Symptom-triggered regimens	Perform the CIWA–Ar every hour to assess the patient’s need for medication Administer one of the following medications every hour when the CIWA–Ar score is at least 8 to 10 points: —Chlordiazepoxide (50–100 milligrams [mg]) —Diazepam (10–20 mg) —Lorazepam (2–4 mg)
Fixed-schedule regimens	Administer one of the following medications every 6 hours: —Chlordiazepoxide (4 doses of 50 mg, then 8 doses of 25 mg) —Diazepam (4 doses of 10 mg, then 8 doses of 5 mg) —Lorazepam (4 doses of 2 mg, then 8 doses of 1 mg) Provide additional medication if these regimens do not control the symptoms (i.e., the CIWA–Ar score remains at least 8 to 10 points)

1 CIWA–Ar = Clinical Institute Withdrawal Assessment for Alcohol, revised.

For further information see figure 1. SOURCE: Mayo-Smith 1997.

Many patients who experience mild withdrawal symptoms do not seek treatment at all. Nevertheless, even those patients may benefit from treatment in the long term, because repeated withdrawal episodes may enhance the brain’s susceptibility to the hyperexcitability that occurs during AW. This process is known as kindling. (For more information on kindling, see the article by Becker, pp. 25–33.) Clinical studies have found that patients with a history of multiple withdrawal episodes have a higher risk of seizures than do patients experiencing their first withdrawal episode (Lechtenberg and Worner 1991). The results of these clinical studies are confounded by differences among the subjects in the severity of dependence, duration of dependence, and quantity of alcohol consumed. The findings are consistent, however, with information obtained using animal research. Thus, prompt appropriate treatment of withdrawal, even in patients with mild symptoms, may conceivably prevent the development of complicated, more severe withdrawal during subsequent episodes.

### Pharmacotherapy of Alcohol Withdrawal Symptoms

Patients who experience more severe withdrawal (i.e., who have CIWA-Ar scores of 8 to 15 or greater) should receive pharmacotherapy to treat their symptoms and reduce their risk of seizures and DT’s. The medications with the best efficacy and safety are the benzodiazepines. Like alcohol, these agents enhance the effect of the neurotransmitter GABA on the brain. Because of their similar effects, benzodiazepines and alcohol are cross-tolerant—in other words, a person who is tolerant to alcohol also is tolerant to benzodiazepines. Cross-tolerance also implies that when a person experiences a deficiency of one agent (e.g., alcohol during withdrawal), the other agent (e.g., a benzodiazepine) can serve as a substitute, thereby easing the withdrawal symptoms.

Benzodiazepines not only improve the symptoms of AW but also reduce the incidence of DT’s and seizures. In addition, they generally are safe and can be administered repeatedly over several hours. The best-studied benzodiazepines for AW treatment are diazepam, chlordiazepoxide, and lorazepam. These agents all are relatively long acting (i.e., for up to several days) and therefore can provide a smooth course of treatment without the risk of rebound symptoms (e.g., seizures) that occur late during withdrawal. Lorazepam should be used in patients with severe liver dysfunction and in patients who are at high risk of experiencing serious medical consequences following sedation, such as people with severe lung disease or elderly patients. Short-acting (i.e., for several hours) benzodiazepines probably are efficacious as well but are associated with a greater risk of rebound symptoms. To prevent recurrence of withdrawal symptoms, these agents must be given in increasingly smaller doses (i.e., require tapering) before they can be discontinued.

**Figure 2:**
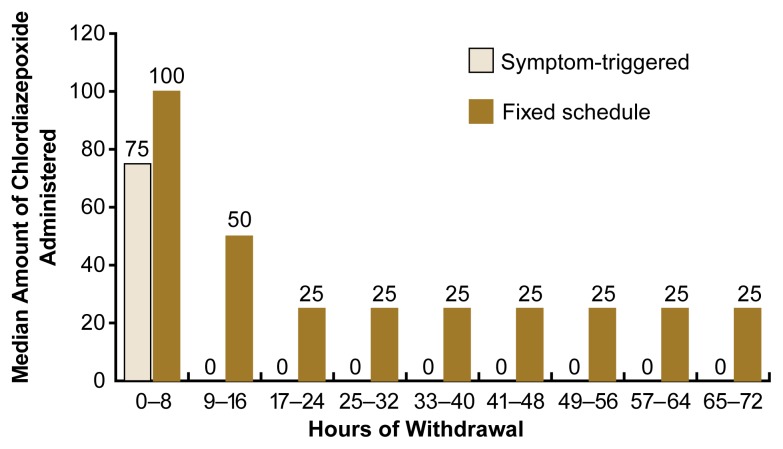
Administration period and median amount of the benzodiazepine chlordiazepoxide administered over the course of alcohol withdrawal to patients undergoing a symptom-triggered or fixed-schedule dosing regimen. The results demonstrate that compared with patients on a fixed-schedule regimen, patients on a symptom-triggered regimen required much less medication for a shorter period of time and were therefore at lower risk for unwanted side effects from the medication. SOURCE: Saitz et al. 1994.

Two primary approaches can be used to administer benzodiazepines during AW treatment: (1) the traditional, fixed-schedule approach and (2) the symptom-triggered approach. In the fixed-schedule dosing regimen, the patient receives a specific dose of the medication every 6 hours for 2 to 3 days, regardless of the presence or severity of symptoms. For the symptom-triggered approach, the patient’s CIWA–Ar score is determined hourly, and the medication is administered only when the score is elevated. Patients with no symptoms receive no medication. This approach is an efficient way to dose benzodiazepines during AW, because it allows the physician to administer the correct amount of medication for the patient’s symptoms. Moreover, when compared with fixed-schedule dosing, symptom-triggered dosing delivers less medication over a shorter period of time (e.g., the first 8 hours of withdrawal) (see figure 2). This approach has not been tested, however, in patients who exhibit no or only mild symptoms but who are at high risk for seizures or whose withdrawal symptoms cannot be adequately assessed. This includes patients who receive general anesthesia or who take medications that block the sympathetic nervous system (e.g., beta blockers, which are used to treat hypertension and other cardiovascular disorders). For these patients, the traditional fixed-schedule dosing regimens may be more appropriate.

Many agents other than benzodiazepines have been used for managing AW. For example, other cross-tolerant medications, such as barbiturates, would be expected to relieve withdrawal symptoms and prevent withdrawal seizures and DT’s. In fact, a few studies have demonstrated that long-acting barbiturates can ease withdrawal symptoms. However, controlled studies have not provided sufficient data to demonstrate that these agents can prevent seizures or DT’s. Furthermore, barbiturates have a narrow therapeutic index—that is, the difference between the minimum dose required for a therapeutic effect and the dose at which the agents become toxic is small.

Alcohol itself also would be expected to improve withdrawal symptoms, and alcoholic patients know that alcohol consumption can relieve their symptoms. Alcohol should not be used, however, to treat withdrawal for several reasons. First, using alcohol as a treatment would promote its acceptability to the alcoholic. Second, alcohol has known toxic effects (e.g., impairing the function of the liver, pancreas, and bone marrow) that are not shared by the safer benzodiazepines. Third, in one clinical study, alcohol was inferior to the benzodiazepine chlordiazepoxide.

Clonidine—an antihypertensive medication—also may have a role in the management of withdrawal symptoms, although it has not been shown to affect the occurrence of withdrawal-specific complications. Another agent that has shown promise for managing AW is the anticonvulsant carbamazepine. Animal studies have demonstrated that the medication may prevent seizures. Moreover, it does not interfere with mental processes, such as learning, whereas other agents (e.g., benzodiazepines) can cause amnesia, mental dullness, and sleepiness (i.e., somnolence). Carbamazepine also does not potentiate alcohol-induced depression of the CNS, nor does it affect respiratory function. In addition, unlike the benzodiazepines, carbamazepine does not have the potential for abuse. Finally, carbamazepine may prevent kindling. This agent has not been shown, however, to prevent withdrawal-specific complications, and it can cause substantial side effects, including nausea and dizziness. (For more information on other medications used in the treatment of withdrawal symptoms, see the article by Myrick and Anton, pp. 38–43.)

Other medications can serve as effective adjuncts to care. For example, beta-blockers (e.g., propranolol and atenolol) can ameliorate some manifestations of withdrawal, such as tachycardia, high blood pressure, and even anxiety, but they increase the likelihood of delirium when used by themselves (i.e., as monotherapy). Consequently, these agents should be used only in combination with benzodiazepines. In general, the use of beta-blockers for treating withdrawal should be considered primarily for patients with coexisting coronary artery disease. Antipsychotic medications such as haloperidol can treat hallucinations and agitation that are unresponsive to adequate doses of benzodiazepines. Because antipsychotic medications can increase the risk of seizures, however, these agents should be used only in combination with benzodiazepines.

### Management of Withdrawal-Specific Complications

AW seizures generally can be prevented by medications that are cross-tolerant with alcohol. For example, benzodiazepines have been shown to prevent both initial and recurrent seizures. Similarly, carbamazepine and the barbiturate phenobarbital probably can prevent AW seizures, although insufficient data exist in humans to confirm this hypothesis. In contrast, phenyotin, an anticonvulsant medication used for treating seizures caused by epilepsy and other disorders, is ineffective for treating AW seizures. Because a diagnosis of AW-related seizures may require further evaluation, however, the agent is sometimes administered until other causes of seizures have been ruled out.

Benzodiazepines also prevent DT’s. However, no known treatments exist to shorten the course of DT’s once this complication has been established. Nonetheless, diazepam can improve outcome by rapidly inducing a calm, awake state, thereby avoiding the traumatic complications associated with severe agitation (Thompson et al. 1975). Constant monitoring is essential for patients experiencing this serious complication.

## Treatment Settings

Traditionally, patients undergoing AW have been treated in hospitals and inpatient alcohol and other drug (AOD) abuse treatment programs. General hospitals and even intensive care units are appropriate for patients whose withdrawal is severe and/or who suffer from comorbid medical, surgical, or psychiatric conditions that require hospitalization. For patients without severe withdrawal or complicating illnesses, inpatient or outpatient AOD treatment settings are appropriate. Some initial reports even indicate that detoxification can be completed successfully in the patient’s home (Stockwell et al. 1991). Withdrawal in settings that offer less intensive monitoring, however, should be considered with caution. For example, as noted previously, the risk factors for more severe withdrawal still need to be better defined. Furthermore, although numerous studies of diverse treatment settings have reported favorable outcomes, many of these studies have included patient groups that were referred specifically to the particular setting being studied. Other studies have included only patients specifically selected for being at low risk for severe withdrawal. Thus, these studies may have been biased toward finding successful outcomes. In the only randomized trial, patients with mild to moderate withdrawal received pharmacological treatment as either inpatients or outpatients (Hayashida et al. 1989). Although outcomes at 6 months did not differ between inpatients and outpatients, fewer outpatients than inpatients completed the treatment and achieved abstinence 1 month later. (For more information on inpatient versus outpatient detoxification, see the article by Hayashida, pp. 44–46.)

## Linking Withdrawal to Alcoholism Treatment

AW is often treated, discussed and studied as an entity distinct from alcoholism treatment. One should remember, however, that withdrawal and its treatment represent a brief period of time (i.e., several hours up to a few days) in the alcoholic’s drinking career. Researchers do not yet know whether the choice of detoxification method has an impact on long-term patient outcomes. For example, one may speculate that early treatment may prevent more serious symptoms during subsequent withdrawal episodes. Furthermore, treatments (both pharmacological and nonpharmacological) that make patients more comfortable may encourage patients to engage in further treatment for their underlying alcohol use disorder and help prevent relapse. Alcoholic patients are at risk for relapse for numerous reasons, including inadequate treatment of their withdrawal symptoms, continued expectations of the rewarding effects of alcohol, and feelings of distress in the absence of alcohol. Effective treatment of withdrawal only addresses the first of these reasons (Dupont and Gold 1995). Nevertheless, for patients who seek assistance with detoxification, treatment of their withdrawal symptoms may present a window of opportunity for initiating alcoholism treatment as well as for attending to other coexisting medical and psychiatric disorders (Samet et al. 1996; Saitz et al. 1997). Accordingly, appropriate recognition and treatment of AW can represent an important, albeit small, first step toward recovery.

## Future Directions

Many unanswered questions remain regarding AW and its management. For example, researchers still must clarify the exact molecular and genetic mechanisms responsible for the varied manifestations of withdrawal. Other studies should address the clinical significance of kindling and the risk factors for more severe withdrawal (Fiellin et al. 1998). Additional research also is needed to determine the most appropriate treatment settings as well as methods of engaging patients in ongoing relapse prevention efforts. Finally, research should investigate techniques to translate knowledge into clinical practice (e.g., ways to improve physician recognition of alcohol dependence) and ways to improve the likelihood that patients receive state-of-the-art, evidence-based treatment. Improved insight into these issues will enable clinicians to improve the efficiency and quality of care for patients who are experiencing or are at risk for withdrawal.

## Footnotes

1 Clinicians generally distinguish between signs and symptoms of a disorder or syndrome. “Signs” are changes in the patient’s condition that can be objectively observed by an examiner (e.g., temperature, a rash, or high blood pressure). Conversely, symptoms are changes that are subjectively perceived by the patient (e.g., irritability or craving for alcohol). The term “manifestations of alcohol withdrawal,” which is used in this article, can refer to either signs or symptoms.

2 The term “hypervigilance” refers to a state of being overly concerned with everything (e.g., one’s surroundings), of being “on edge.” This state manifests itself, for example, by continually looking around and moving one’s head in abrupt, jerky movements.

3 Wernicke’s encephalopathy is an acute condition characterized by general confusion, abnormal eye movements, and difficulty walking or keeping one’s balance.
